# A genomic resource for the sedentary semi-endoparasitic reniform nematode, *Rotylenchulus reniformis* Linford & Oliveira

**DOI:** 10.21307/jofnem-2019-013

**Published:** 2019-04-23

**Authors:** Kurt C. Showmaker, William S. Sanders, Sebastian Eves-van den Akker, Brigitte E. Martin, Roy N. Platt, John V. Stokes, Chuan-Yu Hsu, Benjamin D. Bartlett, Daniel G. Peterson, Martin J. Wubben

**Affiliations:** 1 Department of Crop Science, University of Illinois at Urbana-Champaign, Urbana, IL; 2 Department of Biochemistry, Molecular Biology, Entomology, and Plant Pathology, Mississippi State University, Starkville, MS 39759; 3 Institute for Genomics, Biocomputing, and Biotechnology, Mississippi State University, Starkville, MS 39759; 4 The Jackson Laboratory, Farmington, CT; 5 Department of Plant Sciences, University of Cambridge, Cambridge, UK; 6 Department of Molecular and Cellular Biology, University of Illinois at Urbana-Champaign, Urbana, IL; 7 Department of Genetics, Texas Biomedical Research Institute, San Antonio, TX; 8 Department of Basic Sciences, College of Veterinary Medicine, Mississippi State University, Starkville, MS 39759; 9 Department of Plant and Soil Sciences, Mississippi State University, Starkville, MS 39759; 10 USDA–ARS, Crop Science Research Laboratory, Mississippi State, Starkville, MS 39759

## Abstract

The reniform nematode (*Rotylenchulus reniformis*) is a sedentary semi-endoparasitic species that is pathogenic on many row crops, fruits, and vegetables. Here, the authors present a draft genome assembly of *R*. *reniformis* using small- and large-insert libraries sequenced on the Illumina GAIIx and MiSeq platforms.

The reniform nematode (*Rotylenchulus reniformis* Linford & Oliveira) is a sedentary semi-endoparasitic species that infects 77 plant families, including cotton and soybean, and other high-value crops such as sweet potato and pineapple (Robinson et al., 1997). Here, we report the genome sequencing and assembly of *R. reniformis.*

Biological material for sequencing was a *R. reniformis* stock culture maintained on cotton. Eggs of *R. reniformis* were collected from infected cotton roots (Hussey and Barker, 1973), purified (Jenkins, 1964), surface-sterilized (Baum et al., 2000), pelleted by centrifugation, flash-frozen with liquid nitrogen, and stored at −80°C until DNA extraction. Genomic DNA extraction was performed as described by Blin and Stafford (1976).

Three short-read small-insert libraries (250, 350, and 550 bp) and four large-insert Nextera mate-pair libraries (2-3.5-, 4-6-, 8-10-, and 2-10-kb) were constructed from a pooled population of *R. reniformis* eggs using standard protocols provided by Illumina (San Diego, CA). Additionally, a single *R. reniformis* egg was isolated, its DNA was amplified by whole genome amplification (WGA) using the Qiagen Repli-G single cell kit (Valencia, CA) and short-read small-insert libraries (400- and 600-bp) were constructed and sequenced using standard protocols provided by Illumina (San Diego, CA). Paired-end sequencing was conducted on Illumina GAIIx and Illumina MiSeq systems. We obtained 19.17 Gb and 29.85 Gb from the pooled population short- and long-read libraries, respectively, and 22.29 Gb from the WGA libraries. Pooled population and WGA sequence data was combined for genome assembly.

Mate-pair reads were processed with NextClip (v1.3; Richard et al., 2014) with default parameters to identify fragments containing linker sequence indicating proper circulation and removal of duplicate reads. All libraries were trimmed for adapters and low-quality base calls with Trimmomatic (v0.32; Bolger et al., 2014) and then assembled with ABySS (v1.5.2; Simpson et al., 2009) with a *k*-mer value of 175. The mate-pair libraries were used for scaffolding the assembly with SSPACE (v3.0; Boetzer et al., 2011) followed by gap filling of all libraries with GapFiller (v1.10; Nadalin et al., 2012).

The resultant *R*. *reniformis* genome assembly (RREN1.0, GCA_001026735.1) contained 314 Mb distributed among 100,525 scaffolds with scaffold and contig N50s of 22,705 bp and 5,991 bp, respectively. The genome assembly contained 86.29% of the core eukaryotic proteins utilized by CEGMA (v2.5; Parra et al., 2007). Flow cytometry of propidium iodide stained *R. reniformis* nuclei indicated a genome size of ~190 Mb. The larger genome size (310 Mb) represented by the assembly is likely the result of unresolved haplotypes stemming from heterogeneity within the *R. reniformis* population used for DNA extraction. Repeat prediction with RepeatModeler (Smit and Hubley, 2015) and repeat quantification with RepeatMasker (Smit et al., 2015) identified 245 repeat families within 35.1 Mb of the assembled genome; most elements were DNA transposons or LTR retroelements (20.6 Mb and 11.3 Mb, respectively), while low complexity repeats accounted for 7 Mb. Homologs of known plant-parasitic nematode effector molecules were identiﬁed via tBLASTn and included beta-1–4 endogluconase, chitinase, CLE, CEP, chorismate mutase, invertase, ubiquitin extension protein, and venom allergen-like protein as well as cyst nematode pioneers 4D06, GLAND1, GLAND11, GLAND14, GLAND15, 10C02, 20E03, 33A09, 4G05, 7E05, and 10A06 (reviewed by Mitchum et al., 2013). A variant of the DOG (DOrsal Gland) box DNA motif, an enhancer element associated with dorsal gland effectors (Eves-van den Akker et al., 2016), was identified in the assembly.

GenBank accession numbers: The raw DNA sequence data and genome assembly were deposited at GenBank under BioProject No. PRJNA214681.
